# Access to Psychological Support for Young People Following Stoma Surgery: Exploring Patients’ and Clinicians’ Perspectives

**DOI:** 10.1177/1049732320972338

**Published:** 2020-11-23

**Authors:** Kay Polidano, Carolyn A. Chew-Graham, Adam D. Farmer, Benjamin Saunders

**Affiliations:** 1 School of Medicine, Keele University, Keele, United Kingdom; 2 Midlands Partnership Foundation Trust, Stafford, United Kingdom; 3 University Hospitals of North Midlands NHS Trust, Stoke-on Trent, United Kingdom

**Keywords:** stoma surgery, access to psychological support, chronic illness, qualitative, constructivist grounded theory, United Kingdom

## Abstract

Psychological problems are common among people with inflammatory bowel disease (IBD) following stoma surgery. However, the ways in which stoma-related psychological needs are identified and addressed in health care settings remain unexplored. In this study, we investigated the perspectives of young people with a stoma and health care professionals about access to psychological support. Semi-structured interviews were conducted with young people with an IBD stoma (18–29 years, *n* = 13) and health care professionals (*n* = 15), including colorectal surgeons, gastroenterologists, specialist nurses in IBD and stoma care, and general practitioners in England. Data collection and analysis were informed by constructivist grounded theory. Three analytic categories were developed: “initiating support-seeking,” “affirming psychological needs,” and “mobilizing psychological support,” which capture young peoples’ trajectory to access psychological support. Based on the findings, we highlight the need for both patients and health care professionals to assign greater priority to the identification of psychological symptoms post-stoma surgery. More effective care pathways, which include responsive psychological services, would enhance access to psychological support for young people with a stoma.

## Introduction

Inflammatory bowel disease (IBD) refers to a group of inflammatory conditions affecting the gastrointestinal tract: the most common of which are ulcerative colitis (UC) and Crohn’s disease (CD). Both conditions—which follow a relapse-remitting course—give rise to similar symptoms, including abdominal pain, vomiting, bloody diarrhea, and weight loss. Although medical therapy for IBD has advanced significantly with the advent of biological therapies, stoma surgery may still be necessary for individuals with severe, long-lasting symptoms, or in those who do not respond to medical management. This surgical procedure involves the removal of the inflamed bowel and creation of an opening in the abdomen, through which either the remaining end of the large bowel or end of the ileum (small bowel) is passed. This means that the individual must wear an appliance attached to the surrounding skin, following surgery, into which feces are discharged.

The psychological impact of stoma surgery is well established (Capilla-Díaz et al., 2019; Di Gesaro, 2016; Knowles et al., 2013). A review by Spinelli et al. (2014) highlights that while colectomy with stoma formation may improve quality of life for patients with IBD by inducing remission, the multifaceted challenges of living with a stoma—including body image concerns (Bullen et al., 2012), sexual difficulties (Vural et al., 2016), reduced social functioning (Thorpe & McArthur, 2017), stigmatizing attitudes (Dibley et al., 2020), and lowered self-esteem (Salomé et al., 2014)—may lead to negative emotions and distress. Previous studies have identified high rates of comorbid psychological problems, with 16% to 26% of patients who undergo stoma surgery reporting symptoms of depression and/or anxiety in the immediate postoperative period and also in the long-term (Jayarajah et al., 2016; Wade, 1990). A range of clinical and psychological variables are associated with an increased risk of psychological problems among patients with a stoma—including the planned duration of stoma (i.e., temporary or permanent; de Gouveia Santos et al., 2006; Knowles et al., 2013), development of stoma-related complications (Jayarajah et al., 2016), individual coping style (de Gouveia Santos et al., 2006), and self-efficacy (Bekkers et al., 1996). Thus, guidelines highlight the importance of ongoing monitoring for signs of anxiety and depression in patients following stoma surgery and considering referral for psychological services where appropriate (Association of Stoma Care Nurses UK, 2016; Wound, Ostomy and Continence Nurses Society, 2010).

Despite evidence of negative psychological outcomes following stoma surgery (Capilla-Díaz et al., 2019), a lack of research attention has been afforded to how stoma-related psychological problems are identified and addressed in health care settings. There is a paucity of empirical studies focusing on the provision of psychological care and support for this population: with much of the available evidence coming from reviews, commentaries, and opinion pieces that do not draw on primary data (Black, 2004; Borwell, 2009). Simmons (2008), writing in the context of stoma care services in England, argues that “patients experiencing [psychological] difficulties stay largely undetected, receiving very little, or no, professional support” (p. 21). This also emerges in Notter and Burnard’s (2006) interview-based study conducted with 50 women with an ileostomy. None of their participants, who experienced psychological difficulties after their surgery, reported receiving or being offered any formal counseling or psychological support as part of their postoperative care. Moreover, Knowles et al.’s (2013) small-scale survey study (*n* = 31) showed that despite the high prevalence of depression and anxiety recorded in their Australian sample, very few respondents had received formal support, with the majority (77%) reporting no past or current use of psychological services. This lack of access to psychological services is also reported in the wider IBD literature, suggesting that this issue may be present throughout the care pathway—both before and after stoma surgery. As a result, several researchers have called for an integrated model of care which approaches the treatment of patients with IBD from a holistic perspective: where both their physiological and psychological needs are managed within a collaborative, multi-professional team (Eccles et al., 2020; Mikocka-Walus et al., 2012; Peña-Sánchez et al., 2017). Given the lack of a qualitative evidence-base in the field of IBD and stoma surgery more specifically, no insights are available into the factors influencing access to, and uptake of, psychological support among this patient-group, highlighting a growing need for more qualitative research in this area. However, qualitative studies conducted among different populations with long-term conditions (LTCs), and comorbid psychological problems, suggest that various factors may hinder access to psychological support. These include an over-prioritization given to physical complaints, unavailable or overburdened services, lack of professional confidence in addressing psychological issues, and stigmatizing representations of psychological problems (Carolan & Campbell, 2016; Coventry et al., 2011; Machin et al., 2017; Peyrot et al., 2005).

This evidence gap is particularly pronounced for young people with a stoma, despite many young IBD patients having stoma surgery which leads to unique psychological challenges due to their life stage (Allison et al., 2013; Sinclair, 2009). It is also noteworthy that the majority of the stoma care literature draws exclusively on the perspectives of patients and stoma care nurses, with the views of other health care professionals, consequently, remaining unexplored. The need to consider perspectives that are more varied is especially pertinent in light of evidence suggesting a lack of priority given by gastroenterologists to psychological screening in consultations with their patients with IBD (Mikocka-Walus et al., 2020). The potential of integrated psychological support, however, has not yet been explored in the specific context of patients with an IBD stoma.

These evidence gaps highlight the need for more in-depth exploration of young people’s perceptions of psychological support post-stoma surgery and those of associated health care professionals. This is especially important in light of recent policy directions across various health care systems internationally aimed toward building stronger links between physical and mental health: including the improvement of diagnosis and treatment of mental health problems for people with long-term physical conditions (World Health Organization, 2017). This is the case for the United Kingdom, where the U.K. government’s funded program “Improving Access to Psychological Therapies” (IAPT) was established in 2008, with an aim to deliver large-scale National Health Service (NHS) treatment for common mental health problems, accessed via general practitioner (GP) or self-referral. This service was later expanded to people with physical long-term care conditions and psychological difficulties. Latest reports reveal that 1.6 million referrals to this service were made in the year 2018–2019, suggesting a high demand for psychological treatment in the U.K. population (NHS Digital, 2019). The proportion of those referred peaks at 20 to 24 years of age (Pettit et al., 2017), showing that psychological help-seeking in the general U.K. population is higher among younger people (Cooper et al., 2010).

In this article, we present a qualitative exploration of factors influencing access to psychological support following stoma surgery among young people with IBD (aged 18–29 years): from the perspective of both patients and clinicians. A more complete understanding of the strengths and deficiencies of access to psychological support is essential for the development of interventions and support for this population.

## Materials and Methods

This study adopts a qualitative approach, informed by a constructivist grounded theory methodology (Charmaz, 2014). Constructivist grounded theory evolved from the principles of Glaser and Strauss’ (1967/2017) grounded theory: a methodology based upon systematic techniques aimed at explaining phenomena that are largely unexplored by building theory from empirical observations. While classical grounded theory is rooted in a post-positivist paradigm, privileging objectivity and neutrality in the researcher–participant relationship and analytic process, constructivist grounded theory assumes that individuals construct the realities in which they participate. It hence recognizes that participants will each construct their subjective experiences of stoma care in a different way. Most importantly, constructivist grounded theory analytic techniques allow the development of high-level conceptual insights on access to psychological support, which are grounded in empirical data that have been co-constructed by study participants and the research team.

Ethical approval for the study was granted by the NHS West Midlands–Coventry and Warwick (England) Research Ethics Committee (03/08/17) Ref: 17/WM/0236.

### Sampling and Recruitment

Participants were initially selected using purposive sampling, based on pre-established eligibility criteria. Patients had to be 18 to 29 years of age, have an IBD diagnosis and an ileostomy/colostomy, and be receiving NHS stoma care in England to participate in this study. Health care professionals were eligible if they were directly involved in the care of patients with IBD and/or stoma in an NHS setting in England. Following the selection of the first few participants, principles of theoretical sampling were adhered to, whereby data collection and analysis were conducted concurrently: basing participant selection on the early findings from the analysis (Glaser & Strauss, 1967/2017). In line with this approach, emerging findings helped clarify what data would most usefully add to the emergent theory and which participant characteristics were needed to generate it, thereby increasing the rigor and robustness of findings.

Various methods were used to recruit young people, including an electronic hospital database and a stoma care clinic at a large NHS hospital in the Midlands of England, advertisements by national patient associations, and social media platforms. Health care professionals were recruited via existing clinical networks as well as snowball techniques, where participants were invited to recommend other clinical colleagues who met the eligibility criteria. Participant recruitment was primarily driven by theoretical saturation (Glaser & Strauss, 1967/2017): that is, when all key analytical categories were judged as containing enough breadth and depth, to build solid and robust arguments. As such, we did not try to achieve a predetermined sample size, mainly because it was felt that this was incompatible with the exploratory, inductive aims of the research, reflecting similar recent arguments in the qualitative methods literature (Hammersley, 2015; Saunders et al., 2018). However, recruitment was also influenced by practical considerations. Time limitations for completing the research meant it was only possible to obtain ethical approval to recruit health care professionals from one NHS trust, which led to challenges in recruiting health care professionals, as for some groups, particularly IBD and stoma nurses, there was only a small number from which to recruit. This impacted our ability to achieve sufficient saturation in relation to some of the individual findings within the key analytic categories.

### Data Collection

Semi-structured interviews were conducted by Polidano—who is a social scientist with a medical sociology background—between November 2017 and October 2018. Interviews were considered the most appropriate method for in-depth exploration of participants’ views and experiences about access to psychological support. An interview topic guide was used for each participant group, which was modified over the course of data collection to follow-up on emerging findings. Patient interviews followed a narrative approach to generate detailed accounts, whereas interviews with health care professionals were more focused and directed. Building a strong rapport, especially with patients, was prioritized to reduce power asymmetries and facilitate greater disclosure. Topics covered with both groups included adjustment to stoma surgery, psychological well-being, perceived role of clinicians, professional competency and skills, as well as access to, and adequacy of, psychological services. Examples of questions to patients included the following: “Did you ever seek any form of psychological support following surgery?,” “Talk me through your choices of support-seeking,” “Who do you feel comfortable discussing concerns about mental health form your health care team and why?” Questions to health care professionals included the following: “How would you describe your professional role in the health care of patients with a stoma?,” “To what extent do you feel that supporting the psychological needs of stoma patients falls within your professional remit?,” “What course of action would you take if you suspect that a patient is suffering from any psychological distress related to their stoma?”

Interviews took place at participants’ preferred location, which for young people it included their home, hospital, cafés, or university premises, and for clinicians it included their office or clinic. Written consent was obtained from all participants prior to interviews and re-affirmed verbally at the end of the interview. The average duration of interviews with young people was 55 minutes; for health care professional interviews, the average length was 23 minutes. All interviews were digitally recorded, transcribed, and pseudonymized to protect participants’ identities.

### Data Analysis

Constructivist grounded theory analytic techniques—based on coding, categorization, and constant comparison—were used to analyze the data (Charmaz, 2014), with the aid of the qualitative analysis program NVivo 11. Analysis for both participant groups was conducted separately in the first instance: mapping them onto one another, at a later stage. The first step was for the lead author to read and code each transcript to capture its primary content: starting out with “initial coding,” which was descriptive and characterized by low levels of abstraction, and then proceeding with “focused coding,” which became more selective and conceptual as the analysis progressed. The constant comparison method was employed (Glaser & Strauss, 1967/2017), by comparing data and codes across “young people” cases, across “clinician” cases, and finally among all cases. This led to the conceptualization of codes into higher, more encompassing categories. A sample of four transcripts was independently coded by Chew-Graham, Farmer, and Saunders. The coders were from different disciplinary backgrounds, and the aim of independent coding was therefore to understand cross-disciplinary perspectives on the data and, through discussion, come to an agreement on shared meanings and interpretations. For this reason, it was deemed too simplistic to statistically calculate levels of agreement as a means of assessing reliability, and this was instead achieved in a more nuanced manner through detailed discussion. Three main analytic categories were identified. After the analytical categories were further developed (through cyclical coding and constant comparison), the relationship among them was established. Analytic memos were used throughout this process to record our early interpretations of codes and helping to raise the conceptual level of the analysis. In line with constructivist grounded theory, these conceptual insights were grounded as much as possible in participants’ data, yet they were also influenced to some degree by the disciplinary backgrounds and professional experience of the research team, which consists of two social scientists—whose perspectives are in part influenced by sociological theory on illness experiences—and two academic clinicians—in gastroenterology and general practice, respectively, who provide care for patients with IBD/stomas in their clinical practice. Regular meetings took place during the data analysis process to ensure that a good level of agreement was achieved by the four coders regarding the final analytic categories, and that their influence on the collection and interpretation of data was examined. We outline, below, the characteristics of the participant sample, before reporting the key analytic categories.

## Results

### Participant Characteristics

A total of 13 young people with a stoma (“patients”) and 15 health care professionals were interviewed. The patient sample composed nine females and four males, with an age range of 18 to 29 years (*M* age = 24 years). The clinical characteristics of these patients varied in relation to IBD diagnoses (UC: *n* = 6, CD: *n* = 7), type of stoma (ileostomy: *n* = 11, colostomy: *n* = 2, temporary: *n* = 8, permanent: *n* = 5), and time since stoma surgery which ranged between 2 weeks and 5 years. The health care professional sample includes different professional groups who are all directly involved in the care of patients with IBD and/or stoma: five GPs, four colorectal surgeons, three gastroenterologists, two stoma care nurses, and an IBD nurse. The sample comprises eight males and seven females, with a wide range of experience levels (average: 8 years in current position).

### Principal Findings

The three analytic categories identified in relation to both the patient and health care professional data were as follows:

1. Initiating help-seeking
2. Affirming psychological needs
3. Mobilizing psychological support

While these three categories are distinct, they are also interlinked in that they represent successive junctures in young people’s recollection of their trajectory to seek support. However, as will be explained below, this patient journey was not always seamless and linear due to various challenges encountered along the way. Participants are referred to below using unique study codes to protect their identities. YP denotes “young person” participants whereas HCP refers to “health care professionals.”

#### Initiating support-seeking

All 13 young people suggested that stoma surgery had resulted in improved health status, by inducing long-term resolution of IBD symptoms. The majority of young people welcomed their stoma as a positive life-change, whereas two patients described a struggle to reach acceptance of it, perceiving the stoma as a restriction. Irrespective of their perception of the stoma, emotional distress (characterized by low mood, hopelessness, anger, and at times suicidal ideation) was reported by most young people shortly following surgery: with almost half claiming to have experienced psychological problems in the longer-term, manifesting in terms of low mood and/or anxiety. Daily challenges of living with a stoma—including leakage accidents, body image dissatisfaction and relationship concerns—were generally ascribed as the source of distress (Polidano et al., 2020). However, not all young people who reported psychological problems sought professional help, or had these concerns identified by their health care team. This initial step to seek/receive help appeared to be influenced by various factors:

##### Normalizing psychological problems

Some young people’s reluctance to seek support emanated from the perception that their psychological needs did not warrant professional attention. One participant who struggled to accept his stoma and, consequently, reported feeling distressed, interpreted these negative thoughts and emotions as a normal and justifiable response to his circumstances:

It’s true, I do feel like I’m going crazy sometimes, I feel angry, really angry . . . but what do you expect, really? No one can help me feel better . . . I will get this thing [stoma] reversed and then I will feel better . . . hopefully. (YP, early 20s male, temporary ileostomy)

From this participant’s perspective, distress could not be resolved by reaching acceptance or learning to cope with his stoma, but rather through stoma reversal, which he expected would lead his psychological distress to subside. Given this normalization of distress—seeing it as an understandable reaction to his adverse situation—professional support was regarded as futile and unnecessary. A normalizing attitude toward psychological symptoms was also observed among those patients who viewed their stoma as a positive life-change. In this case, participants appeared to perceive stoma-related psychological problems as a willing compromise for being free from IBD symptoms:

The depression and anxiety I’ve got since surgery, they do bother me [. . .] but I wouldn’t change my stoma for anything in the world. I don’t want to go back to how it was, [as] the stoma changed things for the better. (YP, late 20s female, temporary ileostomy)

However, accepting these psychological problems as part of their “new normal” risked coming at the expense of lacking (perceived) legitimacy to seek professional support:

I: Have you ever talked to someone from your health care team about [your anxiety?]

P: Hmm, no not really. I did discuss my fear of the stoma bag coming off and all that with my stoma nurse, but he doesn’t really know how bad it makes me feel

I: So what stops you then, from telling him?

P: Cause it’s nothing really, if you think about it . . . I don’t want to be complaining all the time [. . .] I know there are worse things which could happen. So I’ll deal with it on my own! (YP, late 20s female, temporary ileostomy)

This young person’s stoic attitude appears to be tied to the meaning she assigns to her psychological needs, which she views as lacking legitimacy in comparison to other possible forms of suffering. This comparison between previous experiences of IBD suffering and current psychological difficulties was drawn frequently by young people:

Overcoming [ulcerative] colitis makes me feel like I can do anything now [. . .] So sure, I have my own struggles but it’s nothing when you think of what I’ve been through. I will take it in my stride and trust that I can handle this. (YP, late 20s female, permanent ileostomy)

The downplaying of current difficulties as well as a preference for self-reliance can be argued to be two downsides of normalizing distress. These two different scenarios suggest that patients’ perception of their psychological needs and how these fit within the context of their past and anticipated future experiences may significantly influence their help-seeking behaviors.

##### Disclosing psychological problems

A lack of information and signposting to support services was reported by young people who wished to seek psychological support, leading them to feel lost trying to navigate the care system:

It’s that offer of saying, ‘look we appreciate that this is a massive change and you’re not maybe going to handle it that well’ and signposting where to find the right support. Currently, when you’re leaving the hospital, you don’t really know where you can access support. [. . .] “You need to tell me . . . who do I speak to if I feel this way?” (YP, late 20s female, permanent ileostomy)

Amid this uncertainty, young people often disclosed their psychological problems to a member of their health care team. While their consultation choices varied, these were largely influenced by the quality of patient–professional relationship. Relational continuity, which is established when patients consult with the same professional over a number of visits, was found to be an important facilitator to disclosure:

I didn’t mind talking to my GP, he’s known me for a long time and is aware of my history [. . .] I wouldn’t feel comfortable discussing certain things with my stoma nurse. I’ve only seen her twice, I barely know her. (YP, mid 20s female, temporary ileostomy)

This participant’s decision to consult her GP was motivated by familiarity and trust, which developed over time. Indeed, GPs were reported to be the most preferred health care professional to consult for psychological needs, followed by IBD nurses and then stoma care nurses. Surgeons and gastroenterologists were not cited by any young person as their choice of health care professional with whom to discuss psychological well-being: largely due to viewing this topic as going beyond their professional remit. This perception was also expressed by surgeons:

[Patients] don’t normally discuss their feelings with me. Probably they would view it more as the role of the specialist nurse and I tend to agree. (HCP, male, colorectal surgeon)

One gastroenterologist added that patients’ reluctance to open up to their consultants might be due to perceived power relationships within the specialist care consultation, making these conversations more intimidating:

Do they open up better to the nurses? They probably do actually. I think sometimes it’s about “oh god, you’re the consultant,” whereas “you’re jus-” . . . “you’re a nurse.” It’s probably less kind of threatening for them. (HCP, female, gastroenterologist)

This perception was also shared by stoma care nurses who emphasized the importance of building a good rapport with patients, viewing this as an investment for facilitating more open conversations about personal issues and disclosure in the future:

There are lots of sides to being a stoma nurse. A lot of it is psychological care, more than physical really. [. . .] So I do tend to spend quite a lot of time psychologically with patients, check how they’re dealing with it. Sometimes, we don’t even look at the stoma, we don’t touch the bag, we just sit and talk. I think this can build up a relationship early. And I think that’s all part of the psychological side of [care] as well, ‘cause [patients] would be more willing to open up if they have a problem or [are] struggling. (HCP, female, stoma care nurse)

Patients’ experiences of how psychological aspects of care were addressed in their stoma care clinic, stand in contrast with how the stoma nurse above reports approaching these issues with patients in her own clinic. Many young people claimed that sparse attention was given to stoma-related psychological needs during follow-up clinics, with physical and practical aspects of stoma care being prioritized instead:

Last time I had an appointment with them, I was literally there for just five minutes; just take the bag off, this is what I think is wrong, this is how we’re going to fix it, put the bag back on, off on your way [. . .] I guess I would have preferred if they touched on psychological issues a little bit. (YP, late 20s female, temporary ileostomy)

The above data extract suggests that time-constraints may be a contributing factor for why psychological needs were not given sufficient attention: with possibly being discouraged from asking about such issues due to not having sufficient time to address them. Awareness of time-pressures faced by stoma care nurses also acted as a deterrent for some young people to bring up their psychological concerns during their clinic, especially when considering the lack of legitimacy that was earlier said to be assigned to these psychological needs:

Sometimes you’re like “Oh I would really love to talk about it” [psychological issues], and then you feel bad for wasting their time, even though it’s not wasting their time, but you kind of feel like “oh it’s not that urgent, I’ll let them go off and see another patient.” (YP, late 20s female, temporary colostomy)

Stoma-related psychological needs appear to be under-prioritized by the patient herself. Her worries about being perceived as a burden for disclosing her psychological issues suggest that, in her eyes, responsibility for psychological care does not fall squarely within stoma care nurses’ professional remit: a perception which starkly contrasts with the views of stoma care nurses in this study.

##### Detection of psychological distress by health care professionals

The process of help-seeking may also be initiated by health care professionals, who may be the ones to detect signs of psychological distress among their patients. However, the extent to which health care professionals viewed this as part of their role varied across professional groups. A common perception held among surgeons and gastroenterologists was that this task pertained specifically to specialist nurses:

Everybody brings different expertise to the multidisciplinary team; there is the surgeon, obviously whose role is to create the stoma, the gastroenterologist to manage the IBD, and then there are the IBD nurses and stoma nurses as well, who among other things, provide the psychological input. (HCP, male, colorectal surgeon)

The identification of psychological distress was recognized by stoma care nurses as an integral part of their professional role, as they reported being alert to the presence of any psychological problems from the first preoperative care meeting with their patients:

I do pre-op counselling for patients and [. . .] I try to pick up from, a psychological point of view, about the patient’s mental health prior to stoma surgery, to see if they’ve already got pre-disposing issues. (HCP, female, stoma care nurse)

While pre-disposing mental health issues prompted stoma care nurses to pay closer attention to psychological adjustment following stoma surgery, the importance of carefully observing patients’ mood as well as subtler cues, such as their handling of stoma bag, was emphasized in all cases:

Sometimes you can pick up on psychological issues [. . .] from the way patients manage their bag. [It] might show you that psychologically, they’re not coping with the stoma. It might be that they’re changing their bag too often and it’s because of something psychological, perhaps they’re too anxious. (HCP, female, stoma care nurse)

The responsibility for identifying psychological needs among this patient-group was also acknowledged by all GPs in the study. Although they reported being seldom consulted for stoma-specific reasons, but more commonly for general health problems, GPs viewed any consultation as an opportunity to explore psychological well-being. This responsibility was framed within a holistic and person-centered approach described as integral to general practice:

It is a core thing to help people manage their kind of physical things alongside their mental health and recognizing that they go together. I would expect people’s mental health to naturally dip after having this type of surgery. Life with a stoma is naturally going to be challenging. And in that age-group [young people] there’s quite a lot of mental health needs anyway, so for me that would be a big concern. I would definitely keep an eye out for the state of their mental health. (HCP, female, GP)

This GP’s awareness about the psychologically challenging nature of stoma surgery, particularly in young people, thus led her to approach consultations with greater vigilance.

#### Affirming psychological needs

In the next phase of the care-seeking trajectory, young people’s psychological needs were assessed by clinicians to determine whether treatment and/or professional support would be required. Among the various professional groups interviewed, GPs expressed the most confidence in undertaking such assessments, largely due to their vast experience in managing mental health needs in their usual clinical practice. Although they acknowledged their lack of specialist knowledge in stoma formation, GPs explained how they would treat this situation as akin to any other life-changing medical event:

I feel very confident dealing with mental health issues across the spectrum of any condition. I don’t see people with a stoma as having any different problems in terms of mental health. Their distress may be related to a specific cause but there are other things that go on in their life. It’s the same as dealing with somebody who is an amputee or struggled with diabetes . . . there’s a common theme for me to address. (HCP, male, GP)

Because GPs considered stoma formation to be a life-changing event—one that may justifiably elicit negative emotional reactions—careful attention was given to only “affirm” those psychological problems deemed as “pathological.” However, making the distinction between clinically significant symptoms and normal adjustment reactions was described as not always straightforward. A person-centered approach was favored in this instance, as health care professionals emphasized the importance of “knowing the patient”:

I use my knowledge of them as a person if I knew them before, ‘cause I knew what they have been like. There are some people who I don’t know before, so I would ask about what they were like before [. . .] This allows me to see whether there is an adjustment issue or it’s something pre-morbid which has been made worse. (HCP, female, GP)

The benefits of relational continuity are highlighted once again, as it allows GPs to better assess their patients’ psychological symptoms within the context of past psychological status. In addition, duration of symptoms and time since stoma surgery were also crucial factors to consider during a psychological assessment:

I speak to some people and it’s really obvious. Some people, they’re coming like a week or two after surgery, and obviously they are distressed but that is understandable, they are low because they’re upset, in a way it’s a natural response to a life-changing event. The classification of how we diagnose depression [is] technically 2 weeks of anhedonia, [but] it’s quite short in this case [. . .] because, obviously, it’s a life-changing event. (HCP, female, GP)

Maintaining flexibility in the diagnosis of psychological disorders was emphasized, given the particular context in which these symptoms manifested. This applies particularly to duration of symptoms because, in the initial weeks or possibly months after stoma surgery, patients could still be in the process of adjusting to and accepting their stoma: with their distress thus classified as a normal reaction, rather than a pathological issue. Although the benefits of drawing such a distinction were recognized by health care professionals, some patients were dissatisfied when early reports of distress were interpreted by clinicians as a normal reaction to stoma surgery:

When I mentioned it to the GP [three weeks after surgery] that I was feeling down about things, apparently that’s “normal” [sarcastic tone]. He didn’t seem too bothered about it. I mean in the past, I’ve been on antidepressants [. . .] I’m assuming that’s what they normally prescribe in this situation. But, they haven’t offered me any sort of counselling either. (YP, mid 20s female, temporary ileostomy)

A dissonance emerges between the professional’s and patient’s interpretation of the problem. While the GP appears to interpret low mood as a normal and understandable reaction to undergoing stoma surgery—expecting this to subside once the patient adapts to her changed circumstances—the patient appears to have perceived this normalizing attitude as dismissive and invalidating.

#### Mobilizing psychological support

This final phase captures how young people with an identified psychological need were supported by the health care system. When severe distress was detected in the stoma care clinic, stoma care nurses highlighted the importance of respecting the limits of their professional expertise—mostly due to a lack of formal training—by referring or signposting patients to health care professionals who have specific expertise in managing mental health issues:

There’s not a lot of training out there for psychological care, it’s just experience really. I think that’s what I pull on all the time, being a nurse for so long [29 years]. [. . .] All the [manufacturing] companies [of stoma care products] do support us, learning-wise, and they do touch on it, but the subject of psychological care is just absolutely huge. [. . .] So, it’s important to say: “I’ve done what I can with you, I think we now need to move on to another service.” (HCP, female, stoma care nurse)

The absence of a clear referral pathway for psychological support as part of the IBD Service, however, made it difficult for secondary care professionals to facilitate their patients accessing support. Various health care professionals reported facing situations where despite wanting to help their patients, there were limited services which could be offered to them:

I have a young lad who previously had a subtotal colectomy and attempted suicide on two occasions because of his stoma. [. . .] For this chap, it’s very, very difficult trying to get that psychological input and I’m being sent all around the houses trying to get it. It’s not incorporated into the care pathway. [. . .] We struggle to gain access to psychological services for IBD patients. (HCP, male, colorectal surgeon)

With respect to this lack of access to psychological services, a distinction was drawn by some secondary care professionals between the care pathway for IBD patients and colorectal cancer patients with a stoma:

I think patients with IBD have almost second-class service . . . we haven’t got access to a psychologist for those patients. If we get a cancer patient, even if it is a stoma-related issue, we’ve got access, so I can refer them to the psychologist and they will see them. [. . .] But we don’t have that access for IBD, but they could have the same mental health issues with regard to a stoma, as a cancer patient can! (HCP, female, stoma care nurse)

According to the stoma care nurse above, this disparity in access to psychological service puts patients with an IBD stoma at a disadvantaged position, though both patient-groups share similar challenges and may require the same level of psychological support. In the absence of these referral pathways, patients were often signposted to their GP in primary care. While GPs emphasized that the course of treatment depended on their assessment of each individual case, the benefits of psychological therapies over pharmacological treatment in these particular circumstances were generally expressed:

People can have definite ideas of what they want. Some people feel that they want a quick fix and yes, medication does play a part, but you have to explain to them that that is not going to make the situation better, especially if their low mood [. . .] stems from surgery. It makes them feel better to actually deal with the situation. So, it would definitely be some kind of [talking therapy] just because it’s [stoma] gonna go on for their life, so they have to find ways how to deal with it. (HCP, female, GP)

The GP’s view that psychological therapy is necessary aligns with views about stoma-related distress being caused by difficulties adjusting to a life-altering surgery. Both counseling and cognitive behavioral therapy (CBT) were viewed as useful ways through which patients can resolve these issues and adjust more successfully to their stoma. GPs explained that if psychological therapy was chosen as the way forward, patients would be signposted to their local primary care mental health service, known as IAPT. Two patients received CBT and counseling, respectively, through this service, with each reporting contrasting views about its effectiveness:

I’ve had CBT twice now. [It] has helped with how I would express myself and those sorts of emotions [. . .] My therapist was so nice. He told me “I’ve done some research on stomas before having this appointment with you.” And I said “wow, that’s nice.” That’s really good that he took the effort to research about it, because I don’t think anybody else would. (YP, late 20s female, temporary ileostomy)

I was seeing a counsellor [. . .] but I didn’t get anything from that. ‘Cause you want someone that knows about stomas, you don’t just want a counsellor that does everything, you want someone that is specialized. (YP, early 20s female, permanent ileostomy)

Despite their different viewpoints, both participants stress the importance for therapists to have IBD- and stoma-related knowledge to tailor the psychological intervention to their psychological needs. This preference led the majority of patients and health care professionals alike, to advocate the need for a specialized psychological service to be embedded in the stoma care pathway of patients with IBD:

I think that ideally, [patients] should benefit from [. . .] access to a clinical psychologist, preferably with an interest in bowel diseases, upon referral by a stoma specialist [. . .]. There is definitely a need for it, and I guess it’s about choosing the patients who need it and those that don’t. But obviously, it all comes down to funding! (HCP, male, colorectal surgeon)

Many secondary care professionals, like the participant above, suggested the implementation of a case-finding and referral system, whereby patients are routinely asked questions about their mental health during follow-up visits. If psychological needs are identified and assessed as warranting formal support, patients would then be referred to a psychological service integrated into the care pathway. Because stoma care nurses were perceived by other professionals as possessing more time, knowledge, and a better rapport with patients, they were viewed as the ideal professional group for asking these case-finding questions. Some health care professionals also recommended this system to be implemented as part of preoperative care, to ensure that individuals who may be at increased risk for experiencing adjustment difficulties following stoma surgery receive additional preparation and psychological support.

## Discussion

This study demonstrates how the psychological needs of young people living with a stoma due to IBD are identified and addressed in health care settings. Our findings show that not all young people who experienced psychological problems following stoma surgery reported to have accessed and/or been offered formal support by their health care team. This is consistent with existing findings reporting low percentages of patients with a stoma to utilize psychological services despite reporting psychological needs (Knowles et al., 2013; Richbourg et al., 2007). However, while the reasons behind this lack of engagement with psychological support were not explored in previous studies, primarily due to their survey design, our qualitative study offers closer insight into the barriers encountered at different stages of the care-seeking trajectory: identified on a patient, professional, and system level.

### Comparison With Existing Literature

#### Identification of psychological problems

In common with the literature on LTCs and comorbid psychological needs (Coventry et al., 2011; Machin et al., 2017; Methley et al., 2017), our findings highlight a perceived lack of legitimacy among patients with an IBD stoma to bring their psychological needs to the attention of their health care team. Dixon-Woods et al.’s (2006) “candidacy framework” provides a useful lens to interpret the impact that this perception may have on access to care. This framework is based on the premise that “candidacy” is essential for patients to request a particular treatment, meaning that they must first recognize their symptoms as needing medical attention or intervention. Such recognition was found to be lacking among some of our participants (at least initially), largely due to the interpretation they assigned to their own distress—with some viewing it as transient and justifiable, and others accepting it as a reasonable price to pay for an improved health status (Polidano et al., 2020). This finding resonates with the claim that normalization of distress is common in the care of patients with a physical LTC (Coventry et al., 2011): as psychological symptoms are interpreted as a normal and understandable response. The reluctance to acknowledge and seek professional support for psychological distress identified in our study has also been reported in the IBD literature more broadly. A preference to cope independently, fear of embarrassment, and concerns about stigma have been cited as emotional barriers to access psychological treatment by patients with IBD (Bennebroek Evertsz et al., 2012; Mikocka-Walus et al., 2020).

Relational aspects of health care consultations, particularly continuity of care, were also found to impact young people’s psychological help-seeking: this being also widely reported in the LTC and health services literature (Carolan & Campbell, 2016; Kravitz et al., 2011; Machin et al., 2017). With relational continuity being considered as the hallmark of primary care (Björkelund et al., 2013), many patients in our study cited GPs as their preferred professional with whom to discuss their psychological concerns: similar to the preference of Richbourg, Thorpe, and Rapp’s (2007) survey respondents. Our findings, however, contrast with those generally reported in the stoma care literature in relation to the quality of relationship between patients and their stoma care nurses. Although positive and supportive relationships have generally been reported in existing studies (Allison et al., 2013; Spiers et al., 2016; Thorpe et al., 2014), young people in this study reported dissatisfaction with such relationships, which in turn acted as a key barrier to the disclosure of psychological needs in the stoma care clinic. It is noteworthy, however, that psychological support was not the focus of these existing studies and may, therefore, account for these different perceptions. This dissatisfaction among patients in our study stemmed from their perception of an overemphasis given by stoma care nurses to the physical and practical aspects of stoma care, with a consequent neglect of psychological care. However, it is important to highlight that patients’ perceptions differ from the views expressed by stoma care nurses in this study, as well as in the wider stoma care literature, where the parity of physical and psychological support has been emphasized (Borwell, 2009). Spiers et al. (2016) highlight the structural factors influencing stoma care nurses’ work in the NHS, such as time and staffing pressures, which may result in a diminished capacity to fulfill the demands of the job. Perceptions of time-constraints in stoma care clinics have also been identified in our study, suggesting that systemic issues may partly contribute to these relational challenges.

Consistent with the views expressed in an earlier work by White and Hunt (1997), and more recently by Di Gesaro (2016), our findings also highlight the need for health care professionals (including stoma care nurses, surgeons, and gastroenterologists) to take on a more active role in the identification of psychological difficulties among patients with a stoma. This is mainly because a reactive approach to the detection of psychological problems has transpired in our study, whereby some professionals were seen to either rely on other members of the health care team to fulfill this task or wait for patients themselves to come forward with their concerns. A similar finding was also reported by Mikocka-Walus et al. (2020) in a qualitative study exploring psychological support needs among people with IBD in the United Kingdom and Australia. In this study, it was similarly reported by both patients and health care professionals that gastroenterologists did not actively screen for psychological symptoms: citing limited time, lack of mental health training as well as fear and discomfort in fulfilling such a task. The passiveness with which stoma-related psychological problems were reported to be approached by health care professionals in this study may result in missed opportunities for identifying distress and offering support to those individuals who are struggling psychologically following stoma surgery, especially in light of the barriers to help-seeking reported by young people. The benefits of implementing case-finding or screening procedures for depression and anxiety in stoma care clinics, at crucial time-points, was called for by health care professionals in our study, reflecting similar findings in the literature (Jayarajah et al., 2016; Simmons, 2008; White & Hunt, 1997).

#### Assessment of psychological problems

A further challenge was noted in relation to the assessment of stoma-related psychological needs because the boundary between a normal adjustment reaction to stoma surgery and a diagnosable disorder was blurred. The difficulty in distinguishing between these psychological states has already been highlighted in the stoma literature (White & Unwin, 1998) and features more generally in studies exploring the management of psychological problems in primary care (Geraghty et al., 2015). Such distinction is also emphasized in the *Diagnostic and Statistical Manual of Mental Disorders* (5th ed.; *DSM-5*; American Psychiatric Association, 2013), clarifying that reactions to losses and common stressors, despite appearing similar, do not constitute a mental disorder (American Psychiatric Association, 2013). Because stoma surgery was regarded as an adverse life event, in line with White and Unwin’s (1998) observation, the GPs in our study tended to lean toward a contextual interpretation of distress, that is, viewing it as a justifiable adjustment reaction. A corollary of normalizing distress, however, is “therapeutic nihilism” where professionals feel that not much can be done to ameliorate patients’ distressful situation, hence offering no intervention (Burroughs et al., 2006). In contrast, a clinical suspicion of “disorder” was based on psychological symptoms, which were more severe and persistent. In such case, the importance of involving a trained mental health professional was acknowledged. We argue, however, that there are various implications for accepting young people’s candidacy for psychological support only when symptoms reach a certain threshold of severity and/or duration. First, this might hamper opportunities for early intervention, possibly resulting in a patient’s mental health to deteriorate before receiving appropriate treatment. Second, not having a person’s distress judged as warranting intervention might be experienced as a delegitimizing experience, as has been reported by one participant in this study. This could have a recursive influence on future help-seeking behavior (Rogers et al., 1999), as patients may feel discouraged to reassert their candidacy, even if their psychological difficulties persist or deteriorate.

#### Referral to psychological services

The final and perhaps most crucial barrier for receiving/offering psychological support is the availability (or lack of) as well as adequacy and acceptability of services. Although stoma care nurses acknowledged the importance of supporting their patients on an emotional and mental level, they concurred with Simmons’ (2008) observation that “current training programmes [for stoma care nurses] are not adequately designed to equip them with the skills necessary to undertake those tasks” (p. 24). Accordingly, the importance of referring/signposting patients to a specialized psychological service was highlighted, although it was added that in their local stoma care service, no referral pathway to psychological treatment was available for patients with IBD, hence, leaving primary care to fill this void. In line with clinical guidelines (National Institute for Health and Care Excellence, 2009), all the GPs in our study concurred that because psychological symptoms are clearly linked to a life event—in this case, stoma surgery—talking therapy is recommended as a first-line treatment. Young people were therefore signposted to IAPT, a general primary care mental health service which is run by the NHS in England, offering evidence-based therapies for common mental health problems (e.g., depression and anxiety) and, more recently, also to those individuals whose psychological problems are comorbid with a physical LTC. Young people’s feedback about the service corresponds with findings from Craven et al.’s (2019) study which explored the expectations of patients with IBD about psychological therapy, where participants preferred their therapists to hold specific knowledge about their condition. Because IAPT are non-specialist psychological services, knowledge of IBD and stoma formation may not always be possessed by therapists, raising concerns about whether therapeutic interventions are relevant or effective to treat stoma-related distress. The need for access to psychological services within the stoma care pathway was, therefore, recognized by patients and health care professionals alike, this aligning with standards for health care services by IBD UK (2019) and the British Society of Gastroenterology consensus guidelines on IBD management in adults (Lamb et al., 2019). A recent national audit also supports these findings, with only 24% of IBD adult services found to provide access to psychological services (Royal College of Physicians, 2018), suggesting that concerns expressed by our participants may extend beyond the study sample. Our participants’ belief that integrating psychological care as part of the standard care for patients with an IBD stoma is consistent with a growing body of literature in which the benefits of integrated and multidisciplinary models of IBD care are highlighted (Mikocka-Walus et al., 2012; Schoultz et al., 2016).

### Strengths and Limitations

This study makes an important contribution to the literature on IBD and stoma care; as to our knowledge, it is the first to have investigated access to psychological support among young people with a stoma. It is also among the first studies in this research area to include the perspectives of a range of health care professionals involved in care of this patient-group, particularly those of GPs which to-date have remained absent in the stoma literature. Alongside the perspectives of patients, including the views of various members of the multi-professional team of patients with an IBD stoma has allowed for a more comprehensive outlook on psychological care and support. This was further facilitated by a multidisciplinary research team (two social scientists and two academic clinicians based in primary and secondary care) who brought different, yet complementary, theoretical and clinical perspectives with which to interpret participants’ data. As recommended by Charmaz (2014), the subjective positionalities of researchers were frequently acknowledged and reflected upon during team meetings, for greater transparency and accountability.

Some limitations are also present in this study. The first relates to the sample’s composition, particularly in terms of the small number of IBD and stoma care nurses. As noted earlier, ethical approval was only obtained for recruiting health care professionals from one NHS Trust, leading to a restricted pool of clinical specialist nurses in IBD and stoma care nurses from which to recruit. While generalizability of findings was not a principal aim of this study, but rather to generate in-depth understanding of participants’ views, arguments based on professional roles and perspectives should be interpreted with caution, as other specialist nurses may have different perspectives. The small within-group sample size also made it difficult to note any gender differences in both the young people’s and health care professionals’ responses. Because previous studies have shown that gender is a pertinent factor when it comes to accessing and providing psychological support and mental health outcomes (Liddon et al., 2018), this could be a useful area to explore in future research. Furthermore, data were collected using single interviews, which did not enable us to capture any changes in young people’s experiences and needs for psychological care over time. Adopting a longitudinal design, by interviewing young people at various time-points after stoma surgery, would have provided a more dynamic and detailed understanding of the care-seeking trajectory. Second, the insights provided by our participants were restricted to care received through the NHS in England. Given differences between countries in terms of health care organization, delivery, training and funding, the transferability of our findings to other contexts should be treated with caution. A crucial difference which warrants particular consideration is access to services, which is free at the point of use in the NHS but requires private health insurance in many other health systems. This could strongly influence people’s decision-making around help-seeking, as well as experiences with referrals and availability of services. Notwithstanding such variations, many of our findings may still have wider resonance with other non-U.K. health systems providing care for patients with an IBD stoma. This is due to broad similarities in stoma care pathways and a similar lack of engagement with psychological services among this patient-group, featuring in previous studies situated across different geographical contexts (Knowles et al., 2013; Nieves et al., 2017; Notter & Burnard, 2006; Richbourg et al., 2007).

### Conclusions and Implications for Practice

On the basis of our findings, it may be concluded that psychological support for young people with a stoma is currently treated as an optional add-on rather than an integral part of the IBD care pathway. Barriers impacting patients’ access to psychological support after stoma surgery in the context of IBD were identified on a patient, professional, and system level: with our findings, therefore, having implications on all these levels. To encourage better evaluation of candidacy among young people, more education and awareness are needed about the psychological difficulties which may arise following stoma surgery and the importance of seeking support. It is also useful for health care professionals to be aware of the possible reluctance by patients with a stoma to disclose their mental health concerns in the clinical setting. This could foster a more proactive approach to the identification of psychological problems, whereby health care professionals inquire more openly and directly about their patients’ mental health, and challenge any unhelpful beliefs about their distress. Better training in mental health for all professional groups involved in the care of this patient-group could also improve the identification and management of these psychological needs. Most importantly, the need for developing more effective care pathways, which include psychological services for patients with an identified need, has also been highlighted. Finally, there is also scope for these findings to guide the development and testing of interventions aimed at enhancing access to psychological support, through improving the identification and management of psychological needs among young people with an IBD stoma.
